# Repeatability and Safety of the Functional Capacity Evaluation-One-Handed for Individuals with Upper Limb Reduction Deficiency and Amputation

**DOI:** 10.1007/s10926-017-9723-0

**Published:** 2017-09-20

**Authors:** S. G. Postema, R. M. Bongers, C. K. Van der Sluis, M. F. Reneman

**Affiliations:** 1 University of Groningen, University Medical Center Groningen, Department of Rehabilitation Medicine, P. O. Box 30001, 9700 RB Groningen, The Netherlands; 2 Center of Human Movement Sciences, University Medical Center Groningen, University of Groningen, Groningen, The Netherlands

**Keywords:** Vocational rehabilitation, Test–retest, Reliability, Limits of agreement

## Abstract

*Purpose* To assess repeatability and safety of the functional capacity evaluation-one-handed (FCE-OH), a FCE-OH individuals, consisting of eight items. *Method* The FCE-OH protocol was administered twice to 23 individuals with upper limb absence (87% male; median age 46 years; median 2 days between sessions). To examine repeatability, test–retest reliability and agreement were assessed with the intraclass correlations coefficient (ICC) and limits of agreement (LoA), respectively. Reliability was considered acceptable when ICC-values were ≥0.75. Widths of LoA of four tests were compared with those of healthy adults. Safety and pain response were assessed with a questionnaire. *Results* After controlling for stability of construct, ICC-values ranged between 0.23 and 0.96, and widths of LoA ranged between 16 and 79%. Intertrial (learning) effects were present in three test items. No serious adverse reactions were reported. A pain response was reported by 30% of the participants. *Conclusion* Good or excellent reliability was observed in five tests, while three items showed poor or moderate test–retest reliability. Interpretation of agreement was possible for four tests, of which three showed widths of LoA similar to those reported in healthy adults. Learning effects were present; therefore, interpretation at the individual level should be performed with care. As the CI of several items were wide, confirmation of results in a larger sample is warranted. Safety was confirmed.

## Introduction

After an amputation of the upper limb, return to work is often an important goal of rehabilitation, as employment is generally beneficial for the individual [1, 2]. However, functional capabilities may have altered due to the amputation and prosthesis use. Also individuals who are born with a transversal reduction deficiency of the upper limb may experience physical limitations due to one-handedness, which may influence their functional capacity. As no instrument was available to assess the functional capacity of individuals with upper limb absence (ULA) in a standardized environment, a functional capacity evaluation (FCE) for one-handed individuals was developed [3]. This instrument can be used to guide decision making of rehabilitation professionals regarding suitable work, or to measure outcomes of vocational rehabilitation programs. Moreover, it helps assessing work limitations due to musculoskeletal complaints, which are a frequent problem in individuals with ULA [4, 5]. Test outcomes can be compared directly with workload or indirectly with reference values [6]. The FCE for one-handed individuals (FCE-OH) contains items that are adapted from an FCE for individuals with work-related upper limb disorders (WRULD) [7]. While all tests of the FCE-WRULD were considered reliable in healthy adults [8], this cannot automatically be assumed for the tests of the FCE-OH in its target patient group. Because the FCE-OH contains substantial alterations with regard to the FCE-WRULD, and is developed for a different patient group, psychometric properties should be examined separately.

It has been demonstrated in healthy workers and patient groups with different chronic pain syndromes that FCE has not led to serious adverse effects, although, a temporary pain increase is common [9, 10]. The safety of FCE application and pain response during and after FCE application in patients with ULA has not been investigated. The aim of this study was to examine the repeatability and safety of the FCE-OH.

## Method

### Setting

Patients were recruited from, and FCE sessions were held at the Prosthetic Center of the Italian Workers’ Compensation Authority (INAIL) in Vigorso di Budrio, Italy.

### Design

Test–retest design; two FCE sessions were held with an interval of at least 24 h apart. A questionnaire on demographics was answered directly after session 1, while 24 h after session 1 the participant was asked to answer several questions about pain response. Stability of construct (e.g. the participant’s self-perceived physical and mental health status being unchanged) was assessed with a questionnaire prior to session 2. The guideline for reporting reliability and agreement study (GRRAS) checklist was followed [11].

### Participants

Potential participants were inpatients from INAIL, who stayed at the centre for several days for prosthetic fitting, repair or training. They were informed of the study by the prosthetic and therapeutic staff and received an information letter from the primary researcher, who invited them to participate in the study. It was made clear that participation was voluntary and rejection of participation would not influence their treatment at the center. Inclusion criteria were: age 18–62 years (official retirement age in Italy); presence of an upper limb reduction deficiency or amputation at or proximal to the carpal level; normal function of the unaffected hand; all seven items of the Italian translation of the physical activity readiness questionnaire [12, 13] were answered negatively, or, when the latter was not the case, if participation was considered safe by a medical doctor. Prosthesis use was not necessary for participation. All patients who met the inclusion criteria were invited. Determination of sample size was based on sample sizes of previous studies on repeatability of FCEs (ranging from 18 to 50; median of 30 participants [8, 10, 14–18]), and availability of participants during the allocated study period.

### Procedures

All sessions were administered by the same tester, who was trained in the standardized FCE-OH procedures. Participants and tester were blinded for the test results of session 1 until the second session was completed. After a general introduction of the sessions, the participant was verbally instructed how to perform each test. Each test was demonstrated by the tester. The participant was also instructed on the four termination criteria: (1) the participant wished to stop one or all tests for whatever reason; (2) the tester deemed it unsafe to continue; (3) the participant’s heart rate was above 85% of his or her age-related maximum [220-age]; or (4) a set time limit or number of repetitions was reached. Delayed onset (muscle) soreness as a result of the FCE-OH was expected and the participants were informed accordingly before signing the informed consent form. To provide safety during the FCE-OH tests, heart rate was monitored continuously with a heart rate monitor.

Six tests were performed, of which two (repetitive reaching test and fingertip dexterity test) were performed with the unaffected limb and prosthetic limb separately, thus making a total of eight test items. The repetitive reaching test with the unaffected limb and prosthetic limb, the fingertip dexterity test with the unaffected limb and the prosthetic limb, and the handgrip strength test with the unaffected limb were each performed three times (referred to as three trials). The tests were performed in a set order: overhead lifting test with a receptacle, overhead lifting test with a 2.0 kg weight (with the unaffected limb), overhead working test, repetitive reaching test (alternating three trials with the unaffected limb and three trials with the prosthetic limb), fingertip dexterity test (alternating three trials with the unaffected limb and three trials with the prosthetic limb), and handgrip strength test (three trials with the unaffected limb). The overhead lifting test with a receptacle and the overhead working test were performed two-handed (unaffected and prosthesis hand), unless the participant had no prosthesis available or had a transhumeral amputation. In that case the test was performed with the unaffected limb only. The fingertip dexterity test could not be performed with a cosmetic prosthesis. Materials, objects and test procedures are presented in Appendix.

Pain response was assessed with self-reported questionnaires. After session 1 participants received an extended version of the pain response questionnaire (PQR) [9] and were asked to answer the first three questions 24 h after finishing session 1. These three questions informed after: (1) whether pain was perceived and, if so, the type of pain (muscular pain, other pain, or a combination of these), (2) whether the participant perceived this pain as being directly caused by the FCE session, and (3) whether the patient had experienced any other physical reaction after the first FCE session. The remaining 14 questions were answered prior to session 2, and assessed stability of construct and presence of pain in the 12 h prior to session 2. If pain was present, the location of pain and the severity of the pain [on an 11 point numeric rating scale from 0 (no pain) to 10 (worst imaginable pain)] was asked. To control for stability of construct of measurement, which is a prerequisite for test–retest reliability analyses [19, 20], changes in mental and physical health (equal, better or worse) compared to the first session, and changes in medication use since the first measurement were recorded. Moreover, it was asked whether the participants had received prosthesis training between sessions, and whether changes were made to the prosthesis (e.g. repair) since session 1. In addition, a questionnaire about demographics was administered.

### Data Analysis

All test scores consisted of continuous variables. For the items of the repetitive reaching test and the fingertip dexterity test, and the hand grip strength test the average of three trials was calculated and used for further analyses. Skewness and kurtosis values divided by their standard error were used to assess distribution of normality of difference between test outcomes of session 1 and 2. If both outcomes were smaller than ±1.96 normal distribution was assumed, and a paired sample *t* test was performed to analyse whether test results of session 1 were significantly different from results of session 2. When the difference was not normally distributed, a Wilcoxon signed-rank test was used, and the median and inter quartile range (IQR) were presented. Differences were considered statistically significant when *p* < 0.05.

Descriptives of the test results during the first and second trial, one-way random intraclass correlation coefficients (ICC) for single measures, and 95% confidence intervals (95% CI) of the ICC-values were computed. ICCs of ≥0.90 were interpreted as excellent, ICCs between 0.75 and 0.90 as good, ICCs between 0.50 and 0.75 as moderate, and ICCs of ≤0.50 as poor [21]. ICC-values of ≥0.75 were considered acceptable [15, 16]. To assess repeatability further, 95% Limits of agreement (LoA) were calculated (mean difference ± 1.96 × standard deviation of mean difference), which represent the size of difference between both measurements; approximately 95% of differences will lie between these LoA [22]. Only changes outside the LoA should be considered as real change [20]. In order to get a global impression of the width of the LoA, a ratio between the LoA and the mean score of session 1 and 2 was calculated [((1.96 × standard deviation of mean difference)/(mean session 1, 2)) × 100%]. When the difference between test outcomes of session 1 and 2 was not normally distributed, the value of 1.96 in the previous two calculations was replaced with the value 2 [22]. Interpretation of LoA is a clinical decision and not a statistical one [22]. Widths of LoA of the overhead lifting test, overhead working test, fingertip dexterity test and handgrip strength test were compared with the widths of LoA found in healthy adults [8]; differences of ≥10% were considered deviant. As it is unknown whether the LoAs found in healthy adults are acceptable, clinically relevant interpretation of (widths of) LoA is not possible.

Test–retest reliability explains the extent to which scores for patients who have not changed are the same for repeated measurement over time [20]. Therefore two analyses were performed; first an analysis including all participants, and second an analysis including only the participants with stability of construct of measurement (e.g. stable functional capacity, in this study determined as unchanged physical and mental health status, and no changes in medical use as measured with the PRQ). Furthermore, in order to assess inter-trial variation a repeated measures one-way ANOVA was performed if a test consisting of multiple trials showed a significant difference in test results between sessions 1 and 2. If Mauchly’s test of sphericity was significant, the Greenhouse-Geisser estimate was reported. To examine whether observed trends of intertrial variation were significant post hoc Bonferroni analyses were performed.

All analyses were performed in IBM Statistics SPSS 22 [23].

## Results

Thirty-two individuals were invited to participate, of which two declined. Therefore, 30 individuals participated, of which 23 performed the FCE-OH protocol twice (completers), with a median time of 47.2 h (IQR: 43.7; 68.0) between sessions. Reasons for seven participants not to perform session 2 (non-completers) were: logistic difficulty to schedule a second session due to time constraints (n = 4), declining for unknown reason (n = 2), and no show (n = 1). Characteristics of the participants are presented in Table 1. Differences between completers and non-completers were small (<10% difference for each variable).

**Table 1:** Characteristics of the participants

	All participants	Participants included in the test–retest analyses
Number of participants	30	23
Gender: male/female (n)	24/6	20/3
Age (years) [median (IQR)]	46.2 (35.2; 54.4)	46.0 (35.2; 55.5)
BMI (kg/m^2^) [median (IQR)]	25.8 (23.4; 28.3)	25.7 (23.7; 27.8)
Marital status [n (%)]		
Single	10 (33.3)	7 (30.4)
Living together with partner	19 (63.3)	16 (69.6)
Divorced or widowed	1 (3.3)	0 (0)
Highest level of education [n (%)]		
Primary school	1 (3.3)	1 (4.3)
Middle or high school	21 (70.0)	15 (65.2)
College or university	8 (26.7)	7 (30.4)
Employed [n (%)]	14 (46.7)	12 (52.2)
Level of amputation (n)		
Transhumeral	5 (16.7)	3 (13.0)
Transradial or wrist disarticulation	25 (83.3)	20 (87.0)
Side of deficiency: left/right (n)	13/17	11/12
Cause of deficiency (n)		
Congenital	1 (3.3)	1 (4.3)
Amputation: trauma	29 (96.7)	21 (95.7)
Amputation of dominant hand^a^ [n (%)]	17 (63.0)	12 (60.0)
Time since amputation (years)^b^ [median (IQR)]	1.7 (1.4; 5.1)	1.7 (1.4; 5.1)
At clinic for first fitting with prosthesis^c^ [n (%)]	6 (20.0)	5 (21.7)
Years of prosthesis use [median (IQR)]^d^	1.5 (1.0; 18.0)^e^	1.5 (1.0; 18.8)
Prosthetic use during FCE-OH testing [n (%)]	19 (63.3)	15 (65.2)
Myoelectric traditional	11 (57.9)	8 (53.3)
Myoelectric with multiarticulating hand	1 (5.3)	1 (6.7)
Body-powered	4 (21.1)	3 (20.0)
(Prototype) cosmetic	3 (15.8)	3 (20.0)

^a^Not included in the calculation of the percentage were two individuals who were ambidextrous before the amputation and one individual with a congenital reduction deficiency

^b^Not included in the analysis was one individual with a congenital reduction deficiency

^c^Individuals who visited the clinic for the first fitting with a prosthesis; therefore these individuals were not experienced with prosthesis use and did not have a prosthesis available

^d^Individuals who visited the clinic for the first fitting with a prosthesis were not included in the analysis

^e^One missing value

### Stability of Construct

Six individuals had prosthesis training between FCE-OH sessions 1 and 2, all but one participant used the same prosthesis during both sessions, and two participants had changes to the prosthesis. In total, six individuals mentioned changed health status at session 2: mental health was better (n = 3), or worse (n = 1), or physical health was better (n = 2), or worse (n = 1). No participants had alterations in medication use between sessions. One participant showed a difference in overhead lifting capacity of 16 kg. During session 2, issues with the prosthesis led to substantial difficulties in lifting performance, as observed by the tester and confirmed by the participant. Therefore, the results of this participant were omitted from the analyses of the overhead lifting test.

### Repeatability

Both primary (with all participants; Table 2) and secondary (participants with changed health status excluded; Table 3) analyses showed acceptable reliability (ICC-values of ≥0.75) for five out of eight items of the FCE-OH, and one item close to the 0.75 threshold of acceptable reliability. Differences between primary and secondary analyses were small and did not influence interpretation of acceptability. Secondary analyses revealed widths of LoA ranging between 16 and 79%. Differences of the widths of LoA of the overhead lifting test, fingertip dexterity test, and handgrip strength test observed in this study and in healthy adults (23, 14, and 20%, respectively) [8] were ≤10%, and thus considered similar. The width of LoA of the overhead working test was wider in this study (79%) compared to the width of LoA in healthy adults (41%) [8].

**Table 2:** Test results of two FCE sessions, and limits of agreement and interclass correlation coefficients between these test results (all participants; n = 9–23)

	n	Mean ± SD or median (IQR) session 1	Mean ± SD or median (IQR) session 2	Mean difference ± SD	95% CI of mean difference	*p*	LoA	Ratio of LoA (%)	ICC (95% CI of ICC)	Interpretation ICC
Overhead lifting test with a container (kg)	22^b^	8.0 (5.8; 12.0)^a^	8.0 (6.0; 10.5)^a^	0.2 ± 1.6	−0.5; 0.9	0.581	−3.1; 3.4	40.3	0.89 (0.77; 0.95)	Good
Overhead lifting test with a 2.0 kg weight (s)	23	34.2 ± 7.4	32.1 ± 6.8	2.1 ± 3.7	0.6; 3.6	0.014*	−5.2; 9.4	22.0	0.83 (0.64; 0.92)	Good
Overhead working test (s)	23	216.0 (127.0; 293.0)^a^	185.0 (112.0; 261.0)^a^	37.8 ± 77.0	6.3; 69.3	0.052	−116.3; 191.8	70.6	0.74 (0.48; 0.88)	Moderate
Repetitive reaching test with unaffected limb (s)	22^c^	51.0 (47.2; 53.3)^a^	46.3 (40.2; 50.4)^a^	6.5 ± 5.6	4.2; 8.9	<0.001**	−4.7; 17.7	23.0	0.59 (0.24; 0.81)	Moderate
Repetitive reaching test with prosthesis (s)	13^d,e^	57.7 (46.2; 64.2)^a^	55.0 (44.2; 60.3)^a^	5.1 ± 5.4	2.1; 8.0	0.003**	−5.7; 15.8	19.1	0.88 (0.65; 0.96)	Good
Fingertip dexterity test with unaffected hand (n)	22^f^	12.8 ± 2.3	13.4 ± 2.0	−0.6 ± 1.1	−1.1; −0.1	0.022*	−2.9; 1.6	17.6	0.83 (0.63; 0.92)	Good
Fingertip dexterity test with prosthesis (n)	9^d,e,g^	3.7 ± 1.2	4.5 ± 1.1	−0.7 ± 1.2	−1.5; 0.0	0.098	−3.1; 1.6	56.6	0.38 (−0.30; 0.81)	Poor
Hand grip strength test (kg)	23	43.6 ± 12.3	44.6 ± 11.8	−0.9 ± 4.4	−2.7; 0.8	0.310	−9.5; 7.6	19.4	0.93 (0.85; 0.97)	Excellent

*kg* kilograms, *s* seconds, *n* number of pins placed

*p < .05

**p < .01

^a^Data is presented as median (IQR), as the difference between test outcomes of session 1 and 2 is not normally distributed. Reasons why n < 23

^b^The participant who lifted a difference of 16 kg was omitted from analysis

^c^One participant did not complete all trials during session 1

^d^Eight participants did not use their prosthesis during testing, as they had a transhumeral amputation (n = 3), or did not have a prosthesis available (n = 5)

^e^Two participants did not perform this test due to sweating of the stump, which would cause twisting of the skin in the socket

^f^One participant declined this test during session 2

^g^Three participants used a cosmetic prosthesis and thus could not perform this test

**Table 3:** Test results of two FCE sessions, and limits of agreement and interclass correlation coefficients between these test results, of individuals with a stable construct (six individuals with changed self-perceived physical and mental health status excluded; n = 6–17)

	n	Mean ± SD or median (IQR) session 1	Mean ± SD or median (IQR) session 2	Mean difference ± SD	95% CI of mean difference	*p*	LoA	Ratio of LoA (%)	ICC (95% CI of ICC)	Interpretation ICC
Overhead lifting test with a container (kg)	17	8.0 (6.5; 12.0)^a^	8.0 (7.0; 11.0)^a^	0.2 ± 1.2	−0.3; 0.8	0.414	−2.2; 2.6	29.4	0.94 (0.84; 0.98)	Excellent
Overhead lifting test with a 2.0 kg weight (s)	17	34.5 ± 8.0	31.2 ± 7.0	2.8 ± 3.8	1.0; 4.6	0.007**	−4.6; 10.3	22.8	0.82 (0.57; 0.93)	Good
Overhead working test (s)	17	205.0 (117.5; 313.5)^a^	171.0 (112.0; 278.0)^a^	40.6 ± 83.0	1.1; 80.1	0.136	−125.5; 206.6	78.5	0.73 (0.40; 0.89)	Moderate
Repetitive reaching test with unaffected limb (s)	16	50.2 (44.2; 52.8)^a^	45.3 (39.2; 50.0)^a^	7.0 ± 5.7	4.2; 9.8	<0.001**	−4.5; 18.5	24.2	0.57 (0.13; 0.82)	Moderate
Repetitive reaching test with prosthesis (s)	9	53.0 (43.0; 64.2)^a^	47.3 (40.2; 59.7)^a^	5.7 ± 6.3	1.6; 9.9	0.017*	−6.9; 18.4	23.4	0.87 (0.57; 0.97)	Good
Fingertip dexterity test with unaffected hand (n)	17	12.8 ± 2.1	13.4 ± 2.0	−0.6 ± 1.1	−1.1; 0.0	0.049*	−2.8; 1.6	17.1	0.82 (0.57; 0.93)	Good
Fingertip dexterity test with prosthesis (n)	6	3.5 ± 1.0	4.6 ± 1.3	−1.1 ± 1.2	−2.1; −0.1	0.073	−3.5; 1.3	58.2	0.23 (−0.58; 0.84)	Poor
Hand grip strength test (kg)	17	41.2 ± 11.1	41.7 ± 11.8	−0.6 ± 3.3	−2.1; 1.0	0.498	−7.0; 5.9	15.6	0.96 (0.95; 0.99)	Excellent

*kg* kilograms, *s* seconds, *n* number of pins placed

*p < 0.05

**p < 0.01

^a^Data is presented as median (IQR), as the difference between test outcomes of sessions 1 and 2 is not normally distributed

Participants performed significantly better during the second session on the repetitive overhead lifting test with the 2.0 kg weight, the repetitive reaching test (both with the unaffected limb and the prosthetic limb), and on the fingertip dexterity test with the unaffected hand (Tables 2, 3). Analyses of intertrial variation are presented in Table 4. As the first trial of the repetitive reaching test was performed slower compared to all following trials an extra analysis was performed, omitting this trial. The ICC-value showed an evident increase (to 0.69, 95% CI 0.32; 0.88), however, the 0.75 level of acceptable reliability was still not reached. The width of LoA changed minimally.

**Table 4:** Intertrial effects of the repetitive reaching test and the fingertip dexterity test with the dominant hand

FCE-OH test item	n	Session 1			Session 2			F-value (df)	*p*
		Trial 1	Trial 2	Trial 3	Trial 4	Trial 5	Trial 6		
Repetitive reaching test with the dominant hand (s)	16	54.8 ± 8.9^2^**^; 3^**^; 4^**^; 5^**^; 6^**	50.3 ± 10.2^1^**^; 5^**^, 6^**	47.9 ± 11.7^1^**	45.8 ± 7.3^1^**	43.8 ± 8.9^1^**^; 2^**	42.5 ± 7.8^1^**^; 2^**	19.2 (2.3)	<0.001**
Repetitive reaching test with the prosthetic hand (s)	9	61.1 ± 17.9^5^*^; 6^*	56.8 ± 18.6	53.4 ± 20.1	52.2 ± 14.1	52.0 ± 13.5^1*^	49.9 ± 13.6^1*^	6.6 (2.0)	0.008**
Fingertip dexterity test with the dominant hand (n)	17	11.6 ± 1.6^2^**^; 5^**^; 6^**	13.4 ± 2.4^1**^	13.3 ± 2.8	12.5 ± 2.4^5^*	13.8 ± 2.2^1^**^; 4^*	13.8 ± 2.0^1^**	8.2 (5)	<0.001**

For both items of the repetitive reaching test the Mauchly’s test of sphericity was statistically significant and therefore the Greenhouse-Geisser estimate was reported. Statistically significant differences between trials are reported in superscript (with numbers referring to trials). Data is presented as mean ± SD. Trial 1–3 were performed during session 1, and trial 4–6 during session 2

*s* seconds, *n* number of pins placed, *df* degrees of freedom

*p < 0.05

**p < 0.01

### Safety

**Fig. 1:**
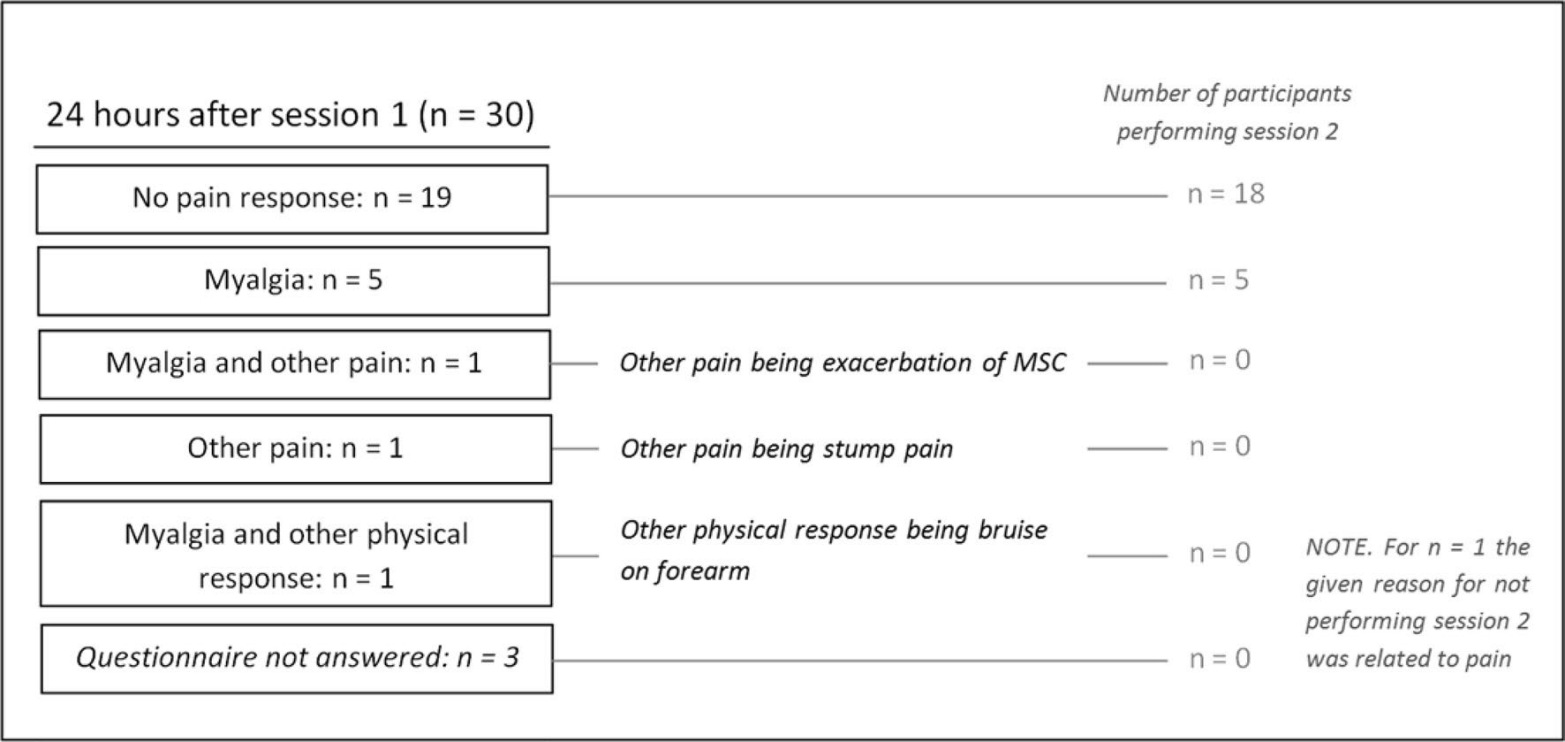
Pain response 24 h after FCE-OH session 1. *MSC* musculoskeletal complaints. Prior to session 2 participants answered a questionnaire regarding the locations of possible complaints. The five individuals with myalgia 24 h after session 1, who performed session 2, had myalgia of the shoulder of the nonaffected limb (n = 1), the shoulder of the affected limb (n = 3) and the forearm of the nonaffected limb and lower back (n = 1)

No tests were terminated due to surpassing 85% of the age-related maximum heart rate. During the first session the overhead lifting test was thrice terminated by the tester, as it was deemed unsafe to continue (generally due to too much bodily swing while lifting, sometimes in combination with difficult grip with the prosthesis). During the second session this occurred only once. No serious adverse reactions occurred, but one individual reported a bruise on the unaffected forearm 1 day after the first session. This adverse reaction was most likely caused by pressure of the lower rim of the container on the forearm, while lifting the container with one hand during the overhead lifting test. After this event the container was padded with foam, and no such incident occurred again. Eight (30%) participants reported a physical response 24 h (pain or other) after the first FCE-OH session, which was partly or completely caused by the test procedure (Fig. 1). Five of these eight individuals performed both sessions; in all five participants the pain was still present at the start of session two (median pain grade: 4, range 2–5). Three of the eight individuals with a pain response did not perform session two; one of these three individuals declined further participation due to the pain response.

## Discussion

### Repeatability

Five of the eight items of the FCE-OH showed acceptable reliability. For the repetitive reaching test with the unaffected limb and the fingertip dexterity test with the prosthesis test–retest reliability was not acceptable. The overhead working test was close to reliable. However, the width of LoA of this test was much wider compared to the width of LoA in healthy adults. Three other tests showed similar widths of LoA, while for four remaining tests comparison of agreement was not possible.

The overhead working test showed a large width of LoA, meaning large within individual differences between sessions. The long duration of the test enhances the chance that an individual performs notably different when repetitively performing the test. However, this test showed considerably smaller widths of LoA when performed by healthy adults [8] and patients with whiplash associated disorders [10] (41 and 49%, respectively, versus 71% in this study), as well as a higher ICC-values (0.90 and 0.83, respectively). The overhead working test is known to show variable ICC-values, ranging from 0.36 in patients with low back pain [16] to 0.90 in healthy adults [8]. Possibly, participants did not completely recover between sessions, and results of this endurance test, were affected by muscle fatigue. Furthermore, it could be that reminiscence of fatigue and possibly pain decreased motivation.

In comparison with the overhead working test, the repetitive reaching test with the unaffected limb was performed significantly better during session 2, which may be caused by learning effects. The higher bound of LoA showed that a test had to be performed at least 19 s faster, to be considered as a real change [20]. The first trial of session 1 was performed significantly slower than all following trials, and therefore it was hypothesized that removing this trial from analysis would improve reliability measures. The ICC-value increased evidently when this trial was removed; however, the 0.75 level of acceptable reliability was not reached. The test showed modest variability between subjects, which can substantially decrease reliability, as reliability demonstrates how well persons can be distinguished from each other, despite measurement error [19, 24]. In the FCE-OH, the repetitive reaching test has been substantially altered and therefore reliability measures cannot be compared with existing literature [3].

For the fingertip dexterity test with the prosthesis the wide CI, possible mediated by the low number of participants, resulted in an uncertain estimate of the ICC-value. No definitive conclusions should be drawn until the reliability of this test is ratified in a larger sample.

Secondary analyses were performed, including only individuals with predefined stability of construct. ICC-values are ratio measures of the between-subject variance and the total variance, the latter including within-subject variance (measurement error) [24, 25]. Excluding individuals without stability of construct decreased within-subject variance, but possibly also the between-subject variance, and definitely the number of participants, which may explain why differences between results of the primary and secondary analyses are small, and did not change interpretation of reliability. Significant difference of test results between session 1 and 2, and inter-trial variation was present in several tests. Nevertheless, when within-participant differences are smaller than between-participant differences, acceptable reliability coefficients are possible [19, 24]. However, it is important to be aware of these effects when assessing a patient.

### Safety

When necessary precautions are taken, the FCE-OH seems to be safe in use, since no serious adverse events occurred, and heart rates of all participants fell within acceptable ranges. One patient experienced a bruise 1 day after FCE-testing, which may be classified as an adverse reaction, which is defined as “any untoward and unintended response to an investigational medical product” [26]. Furthermore, several individuals experienced a pain response after the first FCE-OH session. Pain was mostly denoted as muscular pain and located in the shoulder of the affected limb, which may be caused by the generally passive use of this limb in daily life. Moreover, exacerbation of musculoskeletal complaints may occur. The percentage of individuals with a pain response was much lower than found by Soer et al. [9] (30 vs. 82%, respectively). Reasons for this finding are still speculative and beyond the scope of this article.

### Strengths and Weaknesses

A weakness of the study is that the COSMIN recommendation of 50 participants was not feasible. While the COSMIN guideline recommends 50 participants, the results of this study, and of other FCE reliability studies with a similar number of participants [14, 17, 18], show that a substantially smaller sample can be sufficient to establish reliability. However, the smaller sample may have provoked large CIs of ICC-values, reflecting a general uncertainty about the true ICC, and making it necessary to frame clinical interpretations at the individual level with care. Although results are promising, a study on a larger sample is called for. Most individuals eligible and available for the study were willing to participate; however, completion of both sessions was not always possible and mostly related to time constraints. It is unknown whether this caused any bias.

The interval between both sessions should be long enough to avoid recall bias and fatigue, but short enough to avoid changes in health status, causing genuine difference in performance. Following practical considerations, the interval in this study was variable, with a median of approximately 2 days. This is a shorter time interval compared to most studies, which had time intervals of 1 to 2–3 weeks [8, 10, 16–18], but similar to the time interval in the study of Gross and Battie, who had 2–4 days between sessions [14]. A short interval may cause recall bias of test results (especially for the overhead lifting test, as participants may have recalled the number of weights put in the container), leading to higher ICC-values; but simultaneously may lead to lower ICC-values as the interval might not allow for full recovery. With exception of the overhead working test, we don’t expect the short interval to have played a role, as participants typically performed equal or better during session 2. Reliability measures of the overhead working test are preferably replicated in a study with a larger interval between sessions.

In this study widths or LoA are compared with healthy adults [8]. Some tests of the FCE-OH were substantially altered, and therefore could not be compared. Interpretation of LoA is a clinical decision [22], and a possible way to interpret them is by using the minimal clinically important change. However, the FCE-OH being new, the minimal clinically important change still is to be established. Therefore, further considerations on LoA will follow.

## Conclusion

Good or excellent test–retest reliability was observed in five tests, while the remaining three tests showed poor or moderate test–retest reliability. Comparison of agreement was possible for four tests, of which three showed similar agreement. The FCE-OH was considered safe in use when the right precautions are taken. Large CIs of the ICC-values and LoA, as well as learning effects, make it necessary to frame clinical interpretations at the individual level with care.

## Appendix

See Table 5.

**Table 5:** Materials, objects and procedure of the six FCE-OH tests

Test	Objective	Materials	Procedure	Measurement outcome
Overhead lifting with a container	Functional strength of shoulder and arm musculature	Plastic receptacle (40 × 30 × 26 cm), a wall mounted system with adjustable shelves and weights of 1.0, 2.0 and 4.0 kg	5 lifts from table to crown height v.v. within 90 s in standing position. Increments of weight (2.0 kg for females, 4.0 kg for males) until maximum amount of weight is reached Prosthesis is used when available	Maximum amount of lifted weight (kg)
Overhead lifting with a 2 kg-weight	Functional strength of shoulder and arm musculature	A wall mounted system with adjustable shelves and a weight of 2.0 kg	20 lifts from table to crown height v.v. in standing position, with the unaffected hand	Time needed for 20 lifts v.v. (sec)
Overhead working	Static holding time of shoulder and neck musculature	Aluminium plate adjustable in height with 5 holes, bolts and nuts, and one cuff-weight of 1.0 kg	Standing with hands at crown height, manipulating nuts and bolts, and wearing cuff weights around the unaffected wrist Prosthesis is used when available	Time (s) which static position can be held
Repetitive reaching	Fast repetitive movements of the upper extremity	Two clicking systems [3]	Sitting with clicking systems on wing span, touching clicking system at table height 30 times from left to right v.v. with unaffected or prosthesis hand	Time (s) 30 movements (left to right v.v.) are made
Fingertip dexterity	Fingertip dexterity	Purdue Pegboard (model #32020, Lafayette IN.)	Sitting in front of the pegboard, placing as many pins as possible with unaffected or prosthesis hand in a 30 s trial^a^	Number of pins
Handgrip strength	Isometric hand grip strength	Hand dynamometer (G100 Biometrics^LTD^ E-LINK)	Sitting with the shoulder adducted and neutrally rotated, elbow flexed at approx. 90°, and the forearm and wrist in neutral position, pinching a hydraulic handgrip dynamometer in position 2 for approx. 3 s	Pinched kg

Participants with an above elbow ULA perform all tests without prosthesis

^a^When the test is performed with the prosthesis, the participant is allowed to place the pins in the prosthesis hand with the unaffected hand

## Abbreviations

CI: Confidence interval
FCE-OH: Functional capacity evaluation-one-handed
ICC: Intraclass correlation coefficient
IQR: Interquartile range
LoA: Limits of agreement
ULA: Upper limb absence
WRULD: Work-related upper limb disorders

## References

[1] Waddell G, Burton AK (2006). Is work good for your health and well-being?.

[2] Ross CE, Mirowsky J (1995). Does employment affect health?. J Health Soc Behav.

[3] Postema SG, Bongers RM, Reneman MF, van der Sluis CK (2017). Functional capacity evaluation in upper limb reduction deficiency and amputation: development and pilot testing. J Occup Rehabil.

[4] Østlie K, Franklin RJ, Skjeldal OH, Skrondal A, Magnus P (2011). Musculoskeletal pain and overuse syndromes in adult acquired major upper-limb amputees. Arch Phys Med Rehabil.

[5] Postema SG, Bongers RM, Brouwers MA, Burger H, Norling-Hermansson LM, Reneman MF (2016). Musculoskeletal complaints in transverse upper limb reduction deficiency and amputation in The Netherlands: prevalence, predictors, and effect on health. Arch Phys Med Rehabil.

[6] Soer R, van der Schans CP, Geertzen JH, Groothoff JW, Brouwer S, Dijkstra PU (2009). Normative values for a functional capacity evaluation. Arch Phys Med Rehabil.

[7] Reneman MF, Soer R, Gerrits EHJ (2005). Basis for an FCE methodology for patients with work-related upper limb disorders. J Occup Rehabil.

[8] Soer R, Gerrits EHJ, Reneman MF (2006). Test–retest reliability of a WRULD functional capacity evaluation in healthy adults. Work.

[9] Soer R, Groothoff JW, Geertzen JHB, Van Der Schans CP, Reesink DD, Reneman MF (2008). Pain response of healthy workers following a functional capacity evaluation and implications for clinical interpretation. J Occup Rehabil.

[10] Trippolini MA, Reneman MF, Jansen B, Dijkstra PU, Geertzen JHB (2013). Reliability and safety of functional capacity evaluation in patients with whiplash associated disorders. J Occup Rehabil.

[11] Kottner I, Audigé L, Brorson S, Donner A, Gajeweski BJ, Hróbjartsson A (2011). Guidelines for reporting reliability and agreement studies (GRRAS) were proposed. J Clin Epidemiol.

[12] Shephard RJ (1988). PAR-Q, Canadian Home Fitness Test and exercise screening alternatives. Sports Med.

[13] Thomas S, Reading J, Shephard RJ (1992). Revision of the Physical Activity Readiness Questionnaire (PAR-Q). Can J Sport Sci.

[14] Gross DP, Battié MC (2002). Reliability of safe maximum lifting determinations of a functional capacity evaluation. Phys Ther.

[15] Reneman MF, Dijkstra PU, Westmaas M, Göeken LNH (2002). Test–retest reliability of lifting and carrying in a 2-day functional capacity evaluation. J Occup Rehabil.

[16] Brouwer S, Reneman MF, Dijkstra PU, Groothoff JW, Schellekens JM, Goeken LN (2003). Test–retest reliability of the Isernhagen Work Systems Functional Capacity Evaluation in patients with chronic low back pain. J Occup Rehabil.

[17] Reneman MF, Brouwer S, Meinema A, Dijkstra PU, Geertzen JH, Groothoff JW (2004). Test–retest reliability of the Isernhagen Work Systems Functional Capacity Evaluation in healthy adults. J Occup Rehabil.

[18] James C, Mackenzie L, Capra M (2010). Test–retest reliability of the manual handling component of the WorkHab functional capacity evaluation in healthy adults. Disabil Rehabil.

[19] de Vet HCW, Terwee CB, Knol DL, Bouter LM (2006). When to use agreement versus reliability measures. J Clin Epidemiol.

[20] Mokkink LB, Terwee CB, Patrick DL, Alonso J, Stratford PW, Knol DL (2012). The COSMIN checklist manual.

[21] Innes E, Straker L (1999). Validity of work-related assessments. Work.

[22] Bland JM, Altman DG (1986). Statistical methods for assessing agreement between two methods of clinical measurement. Lancet.

[23] IBM Corp. IBM SPSS Statistics for Windows, Version 22.0. Armonk, NY: IBM Corp; 2013.

[24] Bland JM, Altman DG (1990). A note on the use of the intraclass correlation coefficient in the evaluation of agreement between two methods of measurement. Comput Biol Med.

[25] Altman D, Bland J (1983). Measurement in medicine: the analysis of method comparison studies. Statistician.

[26] Guideline for good clinical practice E6(R1). ICH Harmonised Tripartite Guideline; 1996.

